# Secondary Bacterial Pneumonias and Bloodstream Infections in Patients Hospitalized with COVID-19

**DOI:** 10.1017/ash.2021.85

**Published:** 2021-07-29

**Authors:** Max Adelman, Divya Bhamidipati, Alfonso Hernandez, Ahmed Babiker, Michael Woodworth, Chad Robichaux, David Murphy, Sara Auld, Colleen Kraft, Jesse Jacob

## Abstract

**Group Name:** The Emory COVID-19 Quality and Clinical Research Collaborative

**Background:** Patients hospitalized with COVID-19 are at risk of secondary infections—10%–33% develop bacterial pneumonia and 2%–6% develop bloodstream infection (BSI). We conducted a retrospective cohort study to identify the prevalence, microbiology, and outcomes of secondary pneumonias and BSIs in patients hospitalized with COVID-19. **Methods:** Patients aged ≥18 years with a positive SARS-CoV-2 real-time polymerase chain reaction assay admitted to 4 academic hospitals in Atlanta, Georgia, between February 15 and May 16, 2020, were included. We extracted electronic medical record data through June 16, 2020. Microbiology tests were performed according to standard protocols. Possible ventilator-associated pneumonia (PVAP) was defined according to Centers for Disease Control and Prevention (CDC) criteria. We assessed in-hospital mortality, comparing patients with and without infections using the χ^2^ test. SAS University Edition software was used for data analyses. **Results:** In total, 774 patients were included (median age, 62 years; 49.7% female; 66.6% black). In total, 335 patients (43.3%) required intensive care unit (ICU) admission, 238 (30.7%) required mechanical ventilation, and 120 (15.5%) died. Among 238 intubated patients, 65 (27.3%) had a positive respiratory culture, including 15 with multiple potential pathogens, for a total of 84 potential pathogens. The most common organisms were *Staphylococcus aureus* (29 of 84; 34.5%), *Pseudomonas aeruginosa* (16 of 84; 19.0%), and *Klebsiella* spp (14 of 84; 16.7%). Mortality did not differ between intubated patients with and without a positive respiratory culture (41.5% vs 35.3%; *P* = .37). Also, 5 patients (2.1%) had a CDC-defined PVAP (1.7 PVAPs per 1,000 ventilator days); none of them died. Among 536 (69.3%) nonintubated patients, 2 (0.4%) had a positive *Legionella* urine antigen and 1 had a positive respiratory culture (for *S. aureus*). Of 774 patients, 36 (4.7%) had BSI, including 5 with polymicrobial BSI (42 isolates total). Most BSIs (24 of 36; 66.7%) had ICU onset. The most common organisms were *S. aureus* (7 of 42; 16.7%), *Candida* spp (7 of 42; 16.7%), and coagulase-negative staphylococci (5 of 42; 11.9%); 12 (28.6%) were gram-negative. The most common source was central-line–associated BSI (17 of 36; 47.2%), followed by skin (6 of 36; 16.7%), lungs (5 of 36; 13.9%), and urine (4 of 36; 11.1%). Mortality was 50% in patients with BSI versus 13.8% without (p < 0.0001). **Conclusions:** In a large cohort of patients hospitalized with COVID-19, secondary infections were rare: 2% bacterial pneumonia and 5% BSI. The risk factors for these infections (intubation and central lines, respectively) and causative pathogens reflect healthcare delivery and not a COVID-19–specific effect. Clinicians should adhere to standard best practices for preventing and empirically treating secondary infections in patients hospitalized with COVID-19.

**Funding:** No

**Disclosures:** None

